# An accurate and efficient experimental approach for characterization of the complex oral microbiota

**DOI:** 10.1186/s40168-015-0110-9

**Published:** 2015-10-05

**Authors:** Wei Zheng, Maria Tsompana, Angela Ruscitto, Ashu Sharma, Robert Genco, Yijun Sun, Michael J. Buck

**Affiliations:** Department of Computer Science and Engineering, State University of New York at Buffalo, Buffalo, NY 14203 USA; Center of Excellence in Bioinformatics and Life Sciences, State University of New York at Buffalo, Buffalo, NY 14203 USA; Department of Biochemistry, State University of New York at Buffalo, Buffalo, NY 14203 USA; Department of Oral Biology, State University of New York at Buffalo, Buffalo, NY 14203 USA; Department of Microbiology and Immunology, State University of New York at Buffalo, Buffalo, NY 14203 USA

**Keywords:** Microbiome, Microbiota, Sequencing, MiSeq, 16S rRNA, V1–V3, V3–V4, Mock community

## Abstract

**Background:**

Currently, taxonomic interrogation of microbiota is based on amplification of 16S rRNA gene sequences in clinical and scientific settings. Accurate evaluation of the microbiota depends heavily on the primers used, and genus/species resolution bias can arise with amplification of non-representative genomic regions. The latest Illumina MiSeq sequencing chemistry has extended the read length to 300 bp, enabling deep profiling of large number of samples in a single paired-end reaction at a fraction of the cost. An increasingly large number of researchers have adopted this technology for various microbiome studies targeting the 16S rRNA V3–V4 hypervariable region.

**Results:**

To expand the applicability of this powerful platform for further descriptive and functional microbiome studies, we standardized and tested an efficient, reliable, and straightforward workflow for the amplification, library construction, and sequencing of the 16S V1–V3 hypervariable region using the new 2 × 300 MiSeq platform. Our analysis involved 11 subgingival plaque samples from diabetic and non-diabetic human subjects suffering from periodontitis. The efficiency and reliability of our experimental protocol was compared to 16S V3–V4 sequencing data from the same samples. Comparisons were based on measures of observed taxonomic richness and species evenness, along with Procrustes analyses using beta(β)-diversity distance metrics. As an experimental control, we also analyzed a total of eight technical replicates for the V1–V3 and V3–V4 regions from a synthetic community with known bacterial species operon counts. We show that our experimental protocol accurately measures true bacterial community composition. Procrustes analyses based on unweighted UniFrac β-diversity metrics depicted significant correlation between oral bacterial composition for the V1–V3 and V3–V4 regions. However, measures of phylotype richness were higher for the V1–V3 region, suggesting that V1–V3 offers a deeper assessment of population diversity and community ecology for the complex oral microbiota.

**Conclusion:**

This study provides researchers with valuable experimental evidence for the selection of appropriate 16S amplicons for future human oral microbiome studies. We expect that the tested 16S V1–V3 framework will be widely applicable to other types of microbiota, allowing robust, time-efficient, and inexpensive examination of thousands of samples for population, phylogenetic, and functional crossectional and longitutidal studies.

## Background

Presently, there is growing interest in the study of human microbiota, the consortia of microbes occupying the human body, using 16S rRNA gene sequences as an omnipresent, conserved, and phylogenetically informative housekeeping genetic marker. This recent enthusiasm on dissecting the microbiome has been triggered by important technological advances, and findings supporting that dysbiosis of host-microbe interactions can affect a multitude of human physiological processes [1–3] with some of these interactions being causal [2, 4, 5]. However, our ability to taxonomically characterize the microbiota using sequencing data is still restricted by the lack of universally accepted similarity thresholds [6] and the differential discriminatory power of the nine 16S rRNA hypervariable regions (V1–V9) [7] with no universally accepted region(s) [8]. A number of reports consistently support that amplification of non-representative genomic targets can heavily bias microbiome phylogenetic and diversity studies leading to inconclusive or inaccurate results [7, 9, 10]. Thus, evaluation of the diagnostic power of the targeted genetic markers is essential for accurate identification of specific microbiota.

A commonly used genetic marker for culture-independent characterization of microbial consortia is the 16S rRNA hypervariable V1–V3 region, but its application has been so far limited to protocols involving the Roche/454 pyrosequencing platform (Branford, CT, USA). The latest Illumina sequencing chemistry using the MiSeq has extended the read length to 300 bp, enabling deep profiling of large number of microbiome samples in a single paired-end reaction, and providing similar read lengths to the Roche platform at a fraction of the cost [11]. An increasingly large number of researchers have adopted this technology for the study of various microbiota targeting the 16S rRNA V3–V4 hypervariable region [12, 13]. To expand the applicability of this powerful Illumina MiSeq sequencing platform, we standardized and tested a high-throughput workflow for amplicon library construction and sequencing of the 16S rRNA V1–V3 hypervariable region. Our analysis involved 11 subgingival plaque samples from diabetic and non-diabetic human subjects suffering from periodontitis. The efficiency and reliability of our experimental protocol was compared to 16S V3–V4 sequencing data obtained for the same samples. Comparisons were based on observed taxonomic richness and species evenness, as representative measures of within sample alpha(α)-diversity, along with Procrustes analyses to assess whether beta(β)-diversity estimates are dependent on the hypervariable region used. In addition, we analyzed a total of eight technical replicates for the 16S V1–V3 and V3–V4 regions from a single mock community with known bacterial species and staggered rRNA operon counts, to evaluate the effect of experimental and analytical error on our developed workflow.

Statistical comparison of expected and observed relative species abundances from the mock community clearly supported that our experimental approach provides with an accurate representation of true community composition. Unweighted UniFrac measures of β-diversity for the clinical samples strongly correlated for the two tested 16S rRNA hypervariable regions, demonstrating that both amplicons offer an *overall* similar representation of the human oral microbiota. However, examination of α-diversity for the clinical samples showed that V1–V3 provides with higher phylotype richness, suggesting this region offers with a deeper assessment of population diversity and community ecology for the complex oral microbiota. As current taxonomic interrogation of human microbiota is based predominantly on amplification of 16S hypervariable regions, this study provides researchers with valuable experimental evidence for the selection of appropriate 16S amplicons for future human oral microbiome studies and avoidance of resolution bias. Also, our study highlights the library construction and sequencing conditions necessary for generation of acceptable quality 16S rRNA data. We expect that our tested framework will be widely applicable to other types of microbiota, allowing robust and time-efficient examination of thousands of samples for population genetic, phylogenetic, and functional studies.

## Results and discussion

The new Illumina sequencing 2 × 300 MiSeq platform provides with a high-throughput system for in-depth profiling of microbial consortia from clinical and environmental settings. It is superior compared to previous sequencing chemistries (i.e., Roche/454 pyrosequencing platform; Branford, CT, USA), since it offers the same effective length of reads at a fraction of cost and time. As interest in the study of microbiota has been growing rampantly, many scientists have increasingly adopted the new Illumina MiSeq chemistry to target the 16S V3–V4 region for various microbiome studies [12, 13]. To expand the applicability of this new platform for further population genetic, phylogenetic, and functional microbial studies, we developed a reliable and efficient workflow for amplicon PCR, library construction, and sequencing of the 16S V1–V3 in the MiSeq. Our tested protocol is based on the combination of Illumina overhang sequences with the 16S V1 (27F) forward [14] and V3 (534R) reverse [15] primers (Table 1) and Nextera XT indices, which allow multiplexing of up to 384 samples per MiSeq run.

**Table 1:** Primer sequences used for amplification of the 16S V1-V3 and V3-V4 region

16S region	Name of primer	Primer sequence
V1–V3	Illumina_16S_27F	5′-TCGTCGGCAGCGTCAGATGTGTATAAGAGACAGAGAGTTTGATCMTGGCTCAG
	Illumina_16S_534R	5′-GTCTCGTGGGCTCGGAGATGTGTATAAGAGACAGATTACCGCGGCTGCTGG
V3–V4	Illumina_16S_341F	5′ -TCGTCGGCAGCGTCAGATGTGTATAAGAGACAGCCTACGGGNGGCWGCAG
	Illumina_16S_805R	5′-GTCTCGTGGGCTCGGAGATGTGTATAAGAGACAGGACTACHVGGGTATCTAATCC

To obtain a target coverage depth per sample, someone should consider the minimal number of mappable sequencing reads delivered by the MiSeq (~25 million reads per run) and adjust accordingly the number of multiplexed samples per flow cell. In addition, the clustering density and % PhiX used have an effect on the amount and quality of data generated. We have observed that with the MiSeq 2 × 300 v3 chemistry, lowering the final concentration of 16S sequencing libraries while maintaining a 20 % PhiX decreases the overall data output but increases the percentage of reverse reads with acceptable quality metrics (Q30>70 %, intensities ≥200) and improves the paired-end merging rate. Thus, the final amount of merged paired-end data with acceptable quality is a composite of the number of samples multiplexed, final concentration of sequencing library, and % PhiX control used. The target depth of coverage depends highly on the specific goal of the microbial study. Deeper sequencing is normally required to capture rare taxa in bacterial consortia or to differentiate between similar environmental niches, whereas increasing the depth of coverage does not seem to benefit β-diversity analyses, and efforts should be focused on increasing the number of analyzed samples in these cases [16, 17].

In our experimental workflow, we amplified and sequenced appropriate positive and negative controls, and all reactions were carried out with water and plastic consumables guaranteed as DNA-free. We regard these two precautionary actions as crucial for the accurate and representative characterization of microbial communities, as application of this protocol with uncertified molecular biology reagents in our hands led consistently to contamination with waterborne bacteria. This is in agreement with a growing number of reports highlighting the confounding effects of reagent and laboratory contamination on sequence-based microbiome studies, especially for samples with low microbial biomass (i.e., blood, bronchoalveolar lavage, and others) where contaminating microbial DNA can dominate the bacterial population profile [18-24]. In light of all these findings, we strongly advise the use of controls and reagents free of nucleic acids with our current protocol, especially for samples with limited starting bacterial load. For the latter samples, we also recommend increasing the volume of “amplicon synthesis” reactions to 100 μl (by quadrupling the volumes of required reagents for the first PCR amplification and bead-cleanup) to accurately capture the bacterial composition of these challenging samples.

**Fig. 1:**
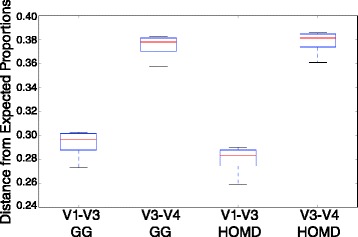
Box plots of Euclidean distances between observed and expected relative species abundances support accurate assessment of true bacterial mock community composition. Euclidean distances between observed and expected relative species abundances of a mock bacterial community were calculated for four technical replicates of the V1–V3 and V3–V4 regions using OTUs picked with the closed-reference protocol against Greengenes (GG) and HOMD. The x-axis represents the different amplicon/OTU picking method used, and the y-axis represents the distance from expected values

A number of publications have highlighted the distorting effects of PCR amplification and sequencing on 16S rRNA-based microbial community profiling even in the presence of routine sequence quality filtering [25], raising the debate over how much of the “rare” microbes are artifacts of experimental procedure [8]. In our study, we used high-proofreading Taq polymerase, stringent quality filtering criteria, and operational taxonomic units (OTU) clustering at 97 % all shown to mitigate overestimation of microbial diversity [25, 26]. In addition, sequencing was based on Illumina chemistry accepted to have lower sequence errors than pyrosequencing, in which homopolymers are the major source of inaccuracy [27, 28]. To further alleviate concerns of PCR bias and sequencing error artificially inflating our diversity estimates and affecting disproportionally one of the tested hypervariable regions, we analyzed a total of eight technical replicates of the 16S V1–V3 and V3–V4 regions from a mock community with known species composition and abundances. Our evaluation was an analysis of qualitative and quantitative taxonomic composition to test if our method accurately measures true community composition while avoiding spurious additional OTUs. Given no experimental error, the observed proportion of OTUs per known bacterial species should align with the expected proportion of the mock community. Our results revealed that our experimental protocol accurately measures the bacterial diversity present in the mock community used (Table 2) with the V1–V3 offering the shortest distance from the expected relative species proportions (Fig. 1).

**Table 2:** Expected and observed relative species abundances of the mock community for the V1–V3 and V3–V4 regions using OTUs picked with two closed-reference protocols

Type strains	Expected relative abundance (%)	Observed relative abundance (%)^a^			
		V1–V3 GG	V3–V4 GG	V1–V3 HOMD	V3–V4 HOMD
*Escherichia coli*	21.91	11.94 (±0.68)	22.60 (±0.61)	15.88 (±0. 72)	22.78 (±0.62)
*Rhodobacter sphaeroides*	21.91	39.07 (±1.57)	12.27 (±0.52)	39.21 (±1.58)	12.33 (±0.52)
*Staphylococcus epidermidis*	21.91	29.93 (±0.92)	0.12 (±0.00)	30.10 (±0.94)	0.24 (±0.02)
*Streptococcus mutans*	21.91	2.96 (±0.39)	20.42 (±0.65)	3.00 (±0.41)	20.49 (±0.65)
*Bacillus cereus*	2.19	0.14 (±0.05)	2.30 (±0.07)	0.15 (±0.05)	2.33 (±0.07)
*Clostridium beijerinckii*	2.19	1.60 (±0.21)	4.01 (±0.16)	1.61 (±0.21)	4.08 (±0.17)
*Pseudomonas aeruginosa*	2.19	2.08 (±0.27)	1.16 (±0.17)	2.10 (±0.28)	1.17 (±0.17)
*Staphylococcus aureus*	2.19	3.39 (±0.19)	30.84 (±1.33)	3.45 (±0.20)	31.38 (±1.33)
*Streptococcus agalactiae*	2.19	1.13 (±0.13)	3.07 (±0.12)	1.14 (±0.13)	3.09 (±0.12)
*Acinetobacter baumannii*	0.22	0.15 (±0.06)	0.31 (±0.03)	0.15 (±0.06)	0.31 (±0.03)
*Helicobacter pylori*	0.22	2.89 (±0.12)	0.67 (±0.05)	2.90 (±0.12)	0.67 (±0.05)
*Lactobacillus gasseri*	0.22	0.01 (±0.01)	0.40 (±0.04)	0.01 (±0.01)	0.40 (±0.04)
*Listeria monocytogenes*	0.22	0.03 (±0.01)	0.20 (±0.01)	0.03 (±0.01)	0.20 (±0.01)
*Neisseria meningitidis*	0.22	0.05 (±0.02)	0.38 (±0.03)	0.05 (±0.02)	0.38 (±0.03)
*Propionibacterium acnes*	0.22	0.03 (±0.01)	0.01 (±0.00)	0.03 (±0.01)	0.01 (±0.00)
*Actinomyces odontolyticus*	0.02	0.00 (±0.00)	0.00 (±0.00)	0.00 (±0.00)	0.00 (±0.00)
*Bacteroides vulgatus*	0.02	0.02 (±0.02)	0.06 (±0.01)	0.02 (±0.02)	0.06 (±0.01)
*Deinococcus radiodurans*	0.02	0.00 (±0.00)	0.03 (±0.00)	0.00 (±0.00)	0.03 (±0.00)
*Enterococcus faecalis*	0.02	0.00 (±0.01)	0.02 (±0.00)	0.00 (±0.01)	0.02 (±0.00)
*Streptococcus pneumoniae*	0.02	0.00 (±0.01)	0.01 (±0.00)	0.00 (±0.01)	0.01 (±0.00)
Others	0	4.56 (±0.13)	1.10 (±0.06)	0.16 (±0.07)	0.01 (±0.00)

^a^Average abundances and standard deviations are calculated based on four replicates

**Fig. 2:**
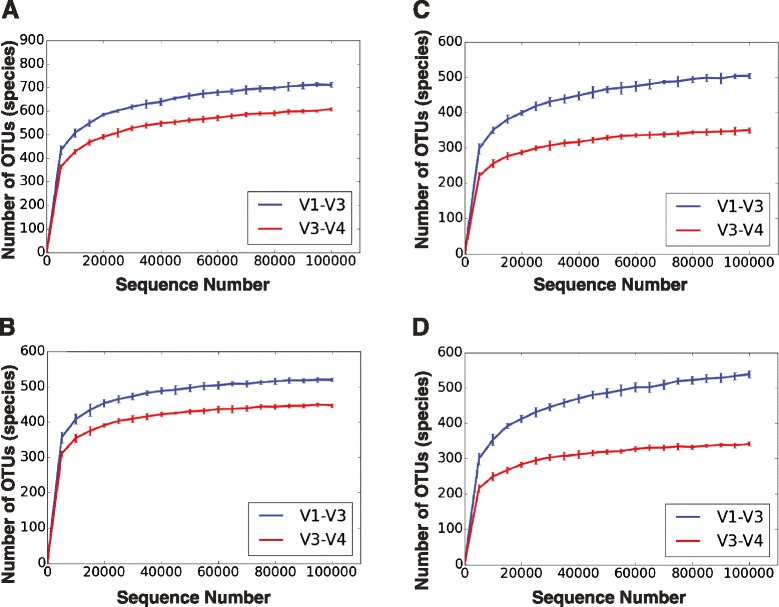
Taxonomic richness is greater for V1–V3 compared to V3–V4 based on four different OTU picking approaches. Alpha rarefaction plots for V1–V3 and V3–V4 hypervariable regions were generated at the species level using the “observed number of OTUs,” a minimum rarefaction level of 1, maximum rarefaction level of 100,001, and a step size of 5,000. Sequence sampling was repeated 10 times for each sample size. OTUs were picked based on the **a** closed-reference OTU picking method against the Greengenes or **b** HOMD database, and **c** the de novo OTU picking with uchime or **d** ChimeraSlayer chimera removal. The x-axis shows the number of sampled sequences, and the y-axis represents the number of observed OTUs. *Red lines* depict taxonomic richness detected using the V3–V4 amplicon, and *blue lines* correspond to the V1–V3 amplicon. *Error bars* exhibit the standard error of mean diversity at each rarefaction level across multiple iterations

The efficiency and reliability of our 16S V1–V3 protocol was also compared to V3–V4 sequencing data obtained from 11 subgingival plaque samples from diabetic and non-diabetic human subjects suffering from periodontitis. We first assigned OTUs to each of the 11 samples, using the closed-reference u-clust OTU picking protocol against the Greengenes and Human Oral Microbiome Database (HOMD) core databases. OTUs were also picked with the de novo protocol using two different chimera-removal approaches. In all approaches, OTU picking was done at the species level using a 97 % sequence similarity level cutoff, after applying strict sequence quality filtering to improve taxonomic accuracy. All employed quality-filtering steps were intended to mitigate the negative effects of PCR-based artifacts and sequencing error on the estimation of population diversity and are integral for accurate dissection of microbial communities [29]. The final number of filtered OTUs obtained with the taxonomy-dependent Greengenes and HOMD approaches, and the de novo with uchime or ChimeraSlayer for the V1–V3/V3–V4 amplicons were 770/654, 543/469, 554/372, and 605/361, respectively. The alpha rarefaction plots generated with the filtered OTU tables from the above picking workflows are shown in Fig. 2. For all OTU picking methods, the V1–V3 tested amplicon captured greater phylotype richness than the V3–V4 region, with V1–V3 detecting 20 to 56 % more OTUs than V3–V4 at the species level.

**Fig. 3:**
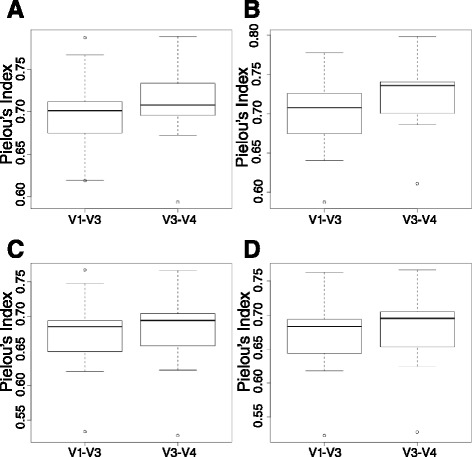
Boxplots of Pielou’s index *J* for V1–V3 and V3–V4 OTUs support a negative relationship between observed oral sample species richness and evenness. Pielou’s index *J* was estimated independently for all generated taxa summaries at the species level to evaluate species evenness detected by the V1–V3 and V3–V4 regions. Analysis was based on OTUs picked based on the **a** closed-reference OTU picking method against the Greengenes or **b** HOMD database, and **c** the de novo OTU picking with uchime or **d** ChimeraSlayer chimera removal. The x-axis shows the value for Pielou’s index *J*, and the y-axis presents the amplicon regions tested. *J* takes values between 0 and 1, with values closer to 1 representing more even quantities of the different species within a community

To compare the phylotype evenness detected by the two amplicons, we estimated Pielou’s index *J* [30] from the species taxonomic summaries generated in QIIME version 1.8.0 using the filtered OTU tables from the closed-reference and de novo approaches for the V1–V3 and V3–V4 regions (Fig. 3). Species evenness is the measure of biodiversity that quantifies how equal a community is numerically [31]. *J* takes values between 0 and 1, with values closer to 1 representing more even quantities of the different species within a community. Overall, for all four OTU picking methods, V1–V3 showed lower phylotype evenness than V3–V4 although none of the pairwise comparisons were statistically significant. The *t* test *P* values for comparisons based on closed-reference against Greengenes, closed-reference against HOMD, de novo with uchime, and de novo with ChimeraSlayer OTU picking methods were 0.60, 0.36, 0.93, and 0.86, respectively. Although the relationship between species richness and evenness can vary in different ecological contexts and has been controversial in the field of ecology [32], more recent empirical studies have shown a significant negative relationship between these two components of diversity [33-35] in agreement with our findings.

**Fig. 4:**
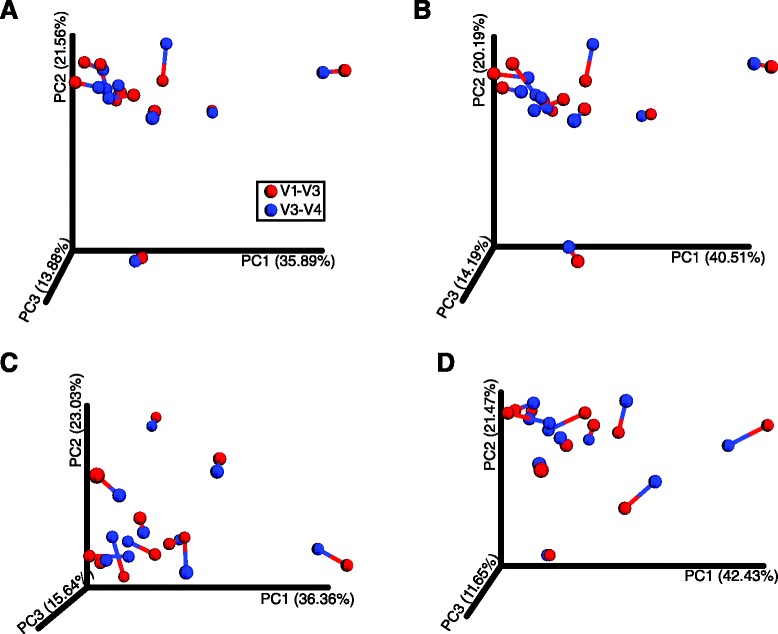
Procrustes analyses demonstrates significant correlation between oral bacterial composition obtained with the V1–V3 and V3–V4 regions. Procrustes analysis of the bacterial composition of V1–V3 (*red*) and V3–V4 (*blue*) regions was calculated using the unweighted UniFrac metric. β-diversity distances were computed at the genus level using the **a** closed-reference OTU picking method against the Greengenes or **b** HOMD database, and **c** the de novo OTU picking with uchime or **d** ChimeraSlayer chimera removal. For a given sample, *red lines* connect to 16S sequence data from the V1–V3 region while *blue lines* connect to points generated from the V3–V4 sequence data. The *M* ^2^ fit reported is from a Procrustes transformation over the first two principal coordinates, while the *P* value is calculated from an empirically determined distribution of *M* ^2^ values over 10,000 Monte Carlo simulations.

To compare the overall differences in population structure across the 11 bacterial communities in relation to the utilized genetic marker (V1–V3 versus V3–V4), we estimated levels of β-diversity at the genus level using the rarefied closed-reference against Greengenes or HOMD and the de novo with uchime or ChimeraSlayer OTU tables at a 40,000 sequencing depth with the unweighted UniFrac distance measure [36, 37]. The generated distance matrices were visualized as three dimensional PCoA plots and statistically compared with Procrustes transformations [38] (Fig. 4). We found that conclusions derived from PCoA plots were independent of the hypervariable region used (*P* = 0.00 for all comparisons), indicating that the tested genetic markers provide with an *overall* similar assessment of the subgingival microbiota.

## Conclusions

In this study, we set out to expand the applicability of the newest Illumina MiSeq powerful sequencing platform by standardizing and testing a high-throughput workflow for amplicon library construction and sequencing of the 16S V1–V3 hypervariable region. V1–V3 data generated with this protocol was compared to V3–V4 sequences generated from the same 11 subgingival plaque samples. Our results demonstrate that our experimental protocol accurately captures true community composition and that both tested amplicons provide an *overall* similar profiling of the human oral microbiota. However, V1–V3 provides with greater phylotype richness and evenness than V3–V4 and thus supports a more representative assessment of population diversity and community ecology for oral bacterial genera. This study provides researchers with valuable experimental insight for the selection of appropriate 16S rRNA amplicons for future human oral microbiome studies and underlines the key experimental and bioinformatics steps necessary for mitigating the effects of bacterial contamination, PCR-based artifacts, and Illumina sequencing error on sequenced-based microbiome analyses. We foresee that the developed workflow will be widely useful to the microbiome community for the high-throughput and accurate and efficient profiling of samples at a reasonable cost. This technology should prove especially useful for time-sensitive microbiome studies involving critical monitoring of environmental or clinical samples at single or multiple timepoints or for pilot microbial studies that could father larger investigations.

## Methods

### DNA extraction, 16S rRNA amplification, library construction, and sequencing

Genomic DNA was extracted from 11 subgingival plaque samples of diabetic and non-diabetic patients with the Fast DNA kit and the FastPrep24-5G instrument according to manufacturer’s recommendations (MP Biomedicals, Santa Ana, CA). Briefly, 200 μl of each oral plaque sample was lysed in 2-ml tubes containing garnet particles and a ceramic sphere with 1 ml of CLS-TC buffer, followed by homogenization in the FastPrep24-5G instrument. Extracted DNA was purified with silica-based spin filters (FastDNA kit) and quantified with the Quant-iT PicoGreen ds DNA Assay Kit (Invitrogen, Eugene, Oregon, USA).

Each DNA sample was subsequently used for 16S amplification of the V1–V3 and V3–V4 hypervariable regions using appropriate negative (UltraClean DNA-free PCR water; MO BIO Laboratories, Inc., Carlsbad, CA, USA) and positive (mouse fecal DNA) controls. Specifically, metagenomic DNA was amplified using the 16S V1 (27F) forward [14] and V3 (534R) reverse [15], and the 16S V3 (341F) forward and V4 (805R) reverse [39] primer pairs with added Illumina adapter overhang nucleotide sequences (Table 1). Amplicon synthesis was performed using thermocycling with 8.5 μl of genomic DNA, 2 μl of amplicon PCR forward primer (2.5 μM), 2 μl of amplicon PCR reverse primer (2.5 μM), and 12.5 μl of 2x KAPA HiFi HotStart Ready Mix (Kapa Biosystems) at 95 °C initial denaturation for 3 min, followed by 25 cycles of 95 °C for 30 s, 62.3 °C for 30 s, and 72 °C for 30 s, and a final extension at 72 °C for 5 min. Reactions were cleaned up with Agencourt AMPure XP beads (Beckman Coulter Genomics) according to the manufacturer’s protocol. Attachment of dual indices and Illumina sequencing adapters was performed using 5 μl of amplicon PCR product DNA, 5 μl of Illumina Nextera XT Index Primer 1 (N7xx), 5 μl of Nextera XT Index Primer 2 (S5xx), 25 μl of 2x KAPA HiFi HotStart Ready Mix, and 10 μl of PCR-grade water (UltraClean DNA-free PCR water; MO BIO Laboratories, Inc., Carlsbad, CA, USA), with thermocycling at 95 °C for 3 min, followed by 8 cycles of 95 °C for 30 s, 55 °C for 30 s, and 72 °C for 30 s, and a final extension at 72 °C for 5 min. Constructed 16S metagenomic libraries were purified with Agencourt AMPure XP beads and quantified with Quant-iT PicoGreen and the KAPA Library Quantification Kit (KAPABIOSYSTEMS). Library quality control was performed with the Agilent Technologies 2100 Bioanalyzer to ascertain quality and average size distribution.

Libraries were normalized and pooled to 4 nM based on qPCR values. Pooled samples were denatured and diluted to a final concentration of 10 pM with a 20 % PhiX (Illumina) control. Sequencing was performed using the MiSeq Reagent Kit V3 in the Illumina MiSeq System. All 22 samples were multiplexed and sequenced in a single lane on the MiSeq using 2 × 300 bp paired-end sequencing. Sequencing reads were generated in less than 65 h. Image analysis and base calling were carried out directly on the MiSeq.

### Mock community

A single aliquot of the microbial mock (synthetic) community B [40], which contains genomic DNA from 20 bacterial strains with staggered ribosomal RNA operon counts (10,000 to 10,000,000 copies per organism per μl), was used in this study. The bacterial strains from which DNA was extracted were *Acinetobacter baumannii*, strain 5377 (NC_009085), *Actinomyces odontolyticus*, strain 1A.21 (NZ_AAYI02000000), *Bacillus cereus*, strain NRS 248 (NC_003909), *Bacteroides vulgatus*, strain NCTC 11154 (NC_009614), *Clostridium beijerinckii*, strain NCIMB 8052 (NC_009617), *Deinococcus radiodurans*, strain R1 (smooth) (NC_001263, NC_001264), *Enterococcus faecalis*, strain OG1RF (NC_017316), *Escherichia coli*, strain K12, substrain MG1655 (NC_000913), *Helicobacter pylori*, strain 26695 (NC_000915), *Lactobacillus gasseri*, strain 63 AM (NC_008530), *Listeria monocytogenes*, strain EGDe (NC_003210), *Neisseria meningitides*, strain MC58 (NC_003112), *Propionibacterium acnes*, strain KPA171202 (NC_006085), *Pseudomonas aeruginosa*, strain PAO1-LAC (NC_002516), *Rhodobacter sphaeroides*, strain ATH 2.4.1 (NC_007493, NC_007494), *Staphylococcus aureus*, strain TCH1516 (NC_010079), *Staphylococcus epidermidis*, FDA strain PCI 1200 (NC_004461), *Streptococcus agalactiae*, strain 2603 V/R (NC_004116), *Streptococcus mutans*, strain UA159 (NC_004350), and *Streptococcus pneumoniae*, strain TIGR4 (NC_003028). All bacterial members of the mock community have completely sequenced genomes and signify a variable range of %GC content and phylogenetic diversity. 16S amplification of the V1–V3 and V3–V4 hypervariable regions, library construction, and sequencing were done as above using 2 μl of genomic DNA for the amplicon synthesis step. Four technical replicates were performed for each hypervariable region.

### Primary 16S rRNA sequence analysis and operational taxonomic unit picking

Primary processing of sequencing reads was done in the context of Quantitative Insights Into Microbial Ecology (QIIME, version 1.8.0) [41, 42]. Initially, demultiplexed paired-end V1–V3 and V3–V4 sequence reads were joined using fastq-join [43, 44] with the default QIIME settings and sequences with ambiguous base calls were removed. Following, primers and barcodes were trimmed from the remaining sequences. Sequencing quality filtering was performed using the FASTX toolkit [45] to isolate sequences having over 90 % base calls with a quality score ≥30.

OTUs were picked separately for the V1–V3 and V3–V4 quality-filtered datasets by taxonomy-dependent (closed-reference based) and taxonomy-independent (de novo) methods. Closed-reference OTU picking was done with QIIME (version 1.8.0, pick_closed_reference_otus.py workflow) [41, 42]. In detail, sequence reads were clustered against a 16S rRNA reference sequence collection with taxonomy annotation using reference-based (uclust_ref) OTU picking [46]. Greengenes (version 13_8) [47] and the HOMD (version 13.2) [48] were used as reference sets separately. Identity thresholds were set to 97 % sequence similarity for species level assignment. Input query reads with hits in the reference sequence collection were given the same taxonomic label as the best hit. Sequences with the same labels were clustered into one OTU. Reads with no hits in the reference sequence collection were excluded from all downstream analyses.

In the de novo OTU picking protocol, reads were clustered based on internal pairwise sequence similarity rather than on an external sequence reference collection using ESPRIT-Tree [49]. The benefit of this de novo OTU picking method is that all reads are clustered and thus novel diversity can be detected. Cutoff thresholds were set to 97 % sequence similarity for species level assignment. Two different chimera identification methods were applied using QIIME version 1.8.0, namely uchime [50] as integrated in the usearch 6.1 [46] pipeline and ChimeraSlayer [51], to identify artifactual amplified sequences originating from multiple parental sequences. PyNAST [52] was used to align representative sequences as input for ChimeraSlayer. The latter uses BLAST [53] to identify potential chimera parents and computes the optimal branching alignment of the query against these two parents. Representative OTUs picked with the de novo protocols were assigned taxonomy using BLAST [53] against the Greengenes (version 13_8) core database and taxonomy. Following, OTU tables representing sample by observation matrices were generated and spurious OTUs (represented by less than 10 sequences) were discarded as a second level of quality filtering. OTUs from clinical samples were aligned using PyNAST [52] against the Greengenes (version 13_8) core database [47], and multiple sequence alignments were used for phylogenetic reconstruction using FastTree [54].

### Comparison of mock community amplicon performance based on expected operon frequencies

The 16S rRNA operon count numbers for the 20 bacterial species of the microbial mock community B [40] were provided from the Bei Resources “Certificate of Analysis for HM-277D” [55]. To compare the reliability of our experimental protocol in recovering the expected structure of the mock community, we computed Euclidean distances between observed and expected relative species abundances of the mock community [56] for the V1–V3 and V3–V4 regions using OTUs picked with the two closed-reference protocols outlined above. Observed relative species abundances were estimated by dividing the observed number of 16S rRNA amplicon reads for each species by the total number of reads per sample.

### Comparison of subgingival plaque sample amplicon performance based on measures of alpha(α)- and beta(β)-diversity

For α-diversity analysis, samples were rarefied multiple times in QIIME version 1.8.0, with a minimum sequence read depth of 1, maximum of 100,001, and a step size of 5,000 reads per sample. Sequence sampling was repeated 10 times for each sample size. Results were plotted as alpha rarefaction plots in QIIME version 1.8.0 using the “observed number of OTUs” metric, to compare true species richness of bacterial communities associated with the two amplicons. Species richness is the measure of α-diversity that is concerned only with the number of species present in a community and not their relative abundance. Taxa summaries were generated for OTUs picked with the closed-reference and de novo protocols using QIIME version 1.8.0 to obtain the relative sample taxonomic abundance at multiple taxonomic levels. Pielou’s index *J* [30] was estimated independently for all generated taxa summaries at the species level using the “evenness” [31] R package to evaluate species evenness detected by the V1–V3 and V3–V4 regions. Species evenness is the second evaluator of α-diversity, which takes into account relative species’ abundances. Obtained V1–V3 and V3–V4 *J* values were statistically compared with the Student’s *t* test using the “t.test” R package [57].

Beta-diversity analysis was computed at the genus level for OTUs picked with each of the four protocols above, using samples rarified to 40,000 reads per sample and the unweighted UniFrac distance measure [36, 37] in QIIME version 1.8.0 (beta_diversity.py). Three dimensional principal coordinate analyses (PCoA) [58] plots were generated using the β-diversity distance matrices by running the principal_coordinates.py script in QIIME. Procrustes transformations (using the script transform_coordinate_matrices.py) [38] were applied in QIIME to compare the compositional structure across the eleven clinical samples with two PCoA plots as input; one built from 16S rRNA V1–V3 sequence data and the other from V3–V4 data. *P* values for Procrustes transformations were generated using 10,000 Monte Carlo simulations by comparing the measure of fit, *M*^*2*^, between matched-sample PCoA plots and the empirically determined distribution of *M*^2^ values. Because *M*^2^ values depend on sample size and data structure, only the generated *P* values were used to compare the Procrustes plots [59]. Results were visualized using Emperor [60] in QIIME (make_emperor.py, −c/ --compare_plots option). Both sets of coordinates were plotted in the same figure, with corresponding points from each tested amplicon connected with red (V1–V3) and blue (V3–V4) lines.

## Abbreviations

PCoA: principal coordinate analysis
PCR: polymerase chain reaction
rRNA: ribosomal RNA
α-diversity: alpha-diversity
β-diversity: beta-diversity
